# Analysis of 255 tracheostomies in an otorhinolaryngology-head and neck surgery tertiary care center: a safe procedure with a wide spectrum of indications

**DOI:** 10.1007/s00405-019-05466-w

**Published:** 2019-05-15

**Authors:** Guanyu Xin, Johanna Ruohoalho, Leif Bäck, Katri Aro, Laura Tapiovaara

**Affiliations:** Department of Otorhinolaryngology-Head and Neck Surgery, University of Helsinki and Helsinki University Hospital, PO Box 263, 00029 HUS Helsinki, Finland

**Keywords:** Airway, Surgical tracheostomy, Mortality, ENT

## Abstract

**Purpose:**

To review indications, patient characteristics, frequency, and safety for surgical tracheostomies performed by otolaryngologist-head and neck surgeons in a single tertiary care center.

**Methods:**

Surgical tracheostomies performed by otolaryngologist-head and neck surgeons at Helsinki University Hospital between January 2014 and February 2017 were retrospectively reviewed. Patient demographics, surgical data, and peri- and postoperative mortality information were collected from the hospital charts. Minimum follow-up was 18 months.

**Results:**

The total population was 255, with a majority (*n* = 181; 71%) of males. The majority of patients (*n* = 178; 70%) were classified as ASA 3 or 4. A total of 198 (78%) patients suffered from head and neck cancer. Multiple (14 altogether) indications for tracheostomy were identified, and simultaneous major head and neck tumor surgery was common (in 58%). Altogether, 163 (64%) patients were decannulated during follow-up with a median cannulation period of 9 days (range 1–425). The surgical mortality was 0.4%.

**Conclusion:**

Simultaneously performed major tumor surgery was the most common indication for a tracheostomy. A notable number of patients had impaired physical status, but relatively insignificant comorbidities. Almost two-thirds of the patients were decannulated during follow-up, although some patients remained tracheostomy dependent for a prolonged period. Tracheostomy was found to be a safe procedure.

**Level of evidence:**

2b.

## Introduction

Tracheostomy is widely considered as one of the most established procedures in surgery [1–5]. Even though less invasive tracheostomy methods, such as percutaneous tracheostomy, have become more common, the traditional open surgical tracheostomy is the method of choice to secure the airway. Severe complications related to tracheostomy are scarce, with approximately 500 tracheostomy-related deaths annually in the USA [6].

The most common indications for tracheostomy range from prolonged mechanical ventilation and trauma to head and neck tumors [7–13], while the main indication is to secure the airway. The utility of tracheostomy and its applications in a tertiary care center vary considerably depending on the population and experience in managing patients requiring this procedure.

Current literature provides limited information about specific details concerning the indications for and frequency of tracheostomies in otolaryngologists’ service as well as patient demographics for those who have required a tracheostomy to secure their airway. The aim of the present study was to offer new insight into these details and to establish a better understanding of the patients who require a tracheostomy and who contemporary otolaryngologists encounter. Thus, we reviewed all patients and the indications and details of the procedures in our tertiary care center during a 38-month period.

## Patients and methods

We reviewed all patients who required a surgical tracheostomy and who were operated by an otolaryngologist-head and neck (ORL-HN) surgeon at Helsinki University Hospital, Helsinki, Finland, between January 2014 and February 2017. The referral area covers over 1.6 million inhabitants. Altogether, 791 patients were identified from the operative database with NCSP (Nordic Classification of Surgical Procedures) codes GBB00 (which stands for tracheostomy) and GBA00 (which stands for tracheotomy). We combined these two codes to stand for the same procedure and screened manually all tracheostomies performed by ORL-HN surgeons. All patients < 18 years (*n* = 17), operations coded incorrectly (concomitant laryngectomy or laryngopharyngectomy; *n* = 5), and patients operated by surgeons other than ORL-HN surgeons (*n* = 514) were excluded from further analysis. The study was approved by the Operative Ethics Committee at the Helsinki University Hospital (DNRO 89/13/03/02C/2011; 13 February 2011) and an institutional research approval was granted (§10, February 6, 30, 2017, HUS).

Patient demographics and surgical details were collected retrospectively from the hospital charts. Patient data included age, sex, body mass index (BMI), American Society of Anesthesiologists (ASA) physical status classification, comorbidities (Adult Comorbidity Evaluation-27) [14], use of anticoagulation, smoking status, alcohol consumption, prior radiotherapy of the neck, and diagnosis. Surgical data included indication for tracheostomy, education level of the head surgeon (consultant or resident), department, the type of anesthesia (local or general), type of surgery (elective or urgent), type of tracheal incision, other possible simultaneous surgery, and time of decannulation. In addition, perioperative and postoperative 30-day mortality was assessed. The minimum follow-up time was 18 months.

Statistical analyses were conducted with SPSS software version 22 (IBM, Armonk, NY, USA). Normality distribution of continuous variables was assessed with visual analysis of histograms and using skewness and kurtosis measures. Continuous variables are reported with median and range, as they are all nonparametric.

## Results

### Baseline demographics

Baseline demographics are presented in Table 1. In total, 255 patients were tracheostomized by ORL-HN surgeons. The majority were male (71%), with a median age of 65 years (range 19–92). A significant proportion of patients were either active smokers (*n* = 119; 47%) or ex-smokers (*n* = 57; 22%). Either active or previous alcohol overconsumption was found in 25% (*n* = 66).

**Table 1:** Demographics of tracheostomized patients

Total (*n* = 255)		Percentage (%)
Age (median, range)	65 (19–92)	
Sex		
Male	181	71.0
Female	74	29.0
BMI (median, range)	23.9 (12–44)	
BMI > 35	10	3.9
High alcohol consumption		
Current	43	16.9
No	127	49.8
Previous	23	9.0
Not known	62	24.3
Tobacco consumption		
Current	119	46.7
No	58	22.7
Previous	57	22.4
Not known	21	8.2
ASA		
1	14	5.5
2	60	23.5
3	116	45.5
4	57	22.4
5	5	2.0
ACE27		
0	95	37.3
1	76	29.8
2	57	22.4
3	27	10.6
Head and neck cancer		
Yes	198	77.6
No	57	22.4

The majority of patients (*n* = 178; 70%) were classified as ASA 3 or 4, thus their present physical status was impaired (median 3). However, the patient cohort did not have major comorbidities, and the majority had ACE-27 score of 0 or 1 (*n* = 95; 37% and *n* = 76; 30%, respectively, median 1, range 0–3). A total of 198 (78%) patients suffered from head and neck cancer (HNC), and 18 (6%) of them had revision tracheostomy.

The indications for tracheostomies are demonstrated in Table 2. Most common indications were related to HNC and its treatment.

**Table 2:** Indications and operative details for patients undergoing tracheostomy

Total (*n* = 255)		Percentage (%)
Indication		
Head and neck oncological surgery		
Included in definitive treatment	147	57.6
Tumor obstruction	31	12.2
Palliation	12	4.7
Infection		
Deep neck infection	16	6.3
Epiglottitis/supraglottitis	16	6.3
Bilateral vocal cord paralysis	17	6.7
Prolonged intubation or intubation failure	8	3.1
Trauma	2	0.8
Other^a^	6	2.4
Access to trachea		
Horizontal	219	85.9
T-type	11	4.3
Lid with downward incisions	10	3.9
Not known	15	5.9
Other simultaneous procedures		
Tumor surgery	90	35.3
Biopsy	58	22.7
Drainage of infection	17	6.7
PEG^c^-tube placement	15	5.9
Other^b^	11	4.3
No other procedure	64	25.1
Urgency		
Elective	141	55.3
Urgent	114	44.7
Surgeons’ educational level		
Consultant	160	62.7
Resident	95	37.3
Anesthesia		
Local	172	67.5
General	83	32.5

^a^Angioedema *n* = 1, cricoid chondritis *n* = 1, subglottic stenosis caused by granulomatous infection *n* = 1, postoperative hemorrhage after tongue base surgery *n* = 1, respiratory failure caused by ALS *n* = 1, subglottic edema caused by congestive heart failure *n* = 1

^b^Hypopharyngoscopy *n* = 2, hemostasis *n* = 2, laryngeal trauma *n* = 1, thyroidectomy *n* = 1, bronchoscopy *n* = 1, cochlea implantation *n* = 1, tonsillectomy *n* = 1, nasogastric tube placement *n* = 1, tooth extraction *n* = 1

^c^Percutaneous endoscopy gastrostomy

### Length of tracheostomy-dependence

We present details of the duration of cannulation in Table 3. The tracheostomy was eventually removed from 163 (64%) patients during follow-up. The majority of them (*n* = 70; 43%) were decannulated between the first and seventh postoperative days. In decannulated patients, the cannulation period ranged from 1 to 425 days, with a median of 9 days. The majority of patients who remained cannula dependent (*n* = 83; 90%) had HNC. The status of the patients at the end of the follow-up is presented in Table 4.

**Table 3:** Time period of cannulation

	Total (*n* = 255)	Percentage (%)
Decannulated^a^		
Yes	163	63.9
No	92	36.1
Median days of cannulation (range)	9 (1–425)	
Days tracheostomized		
1–7	70	27.5
8–30	59	23.1
31–60	12	4.7
61–100	5	2.0
Over 100	17	6.7

^a^Cannulation status at the end of follow-up ( ≥ 18 months)

**Table 4:** Status of all patients (*n* = 255) at the end of the follow-up

Status	(*n* = 255)	Percentage (%)
Alive	154	60.4
Tracheostomized	23	9.0
HNC^a^	19	7.5
Decannulated	131	51.4
HNC	85	33.3
Dead	101	39.6
Tracheostomized	69	27.1
HNC	64	25.1
Decannulated	32	12.5
HNC	30	11.8

*HNC* head and neck cancer

^a^12 underwent subsequent laryngectomy or laryngopharygectomy

### Other findings

Operative details are displayed in Table 2. The majority (86%) of tracheal incisions were horizontal. In 25% of patients, tracheostomy was the only performed procedure, while simultaneous additional surgery was most often related to neoplasms. Urgent tracheostomies were required in 45% of patients.

### Perioperative and postoperative mortality

Four patients (1.6%) died within 30 postoperative days. One patient (0.4%) had extremely difficult anatomy due to multiple HNC recurrences, surgery and adjuvant oncological treatment, resulting in perioperative loss of airway for 14 min, resuscitation and anoxic brain injury. She died on the fifth postoperative day. Her death was considered to result from a surgical complication. One death was associated with patient selection: the patient suffered from a neurological disease that had caused bilateral vocal cord paralysis, which was the indication for tracheostomy. He was restless and agitated postoperatively and removed the cannula repeatedly. Ultimately, attempts to reinsert the cannula failed and the patient died on the 14th postoperative day. Two of the additional deaths were not directly related to the tracheostomy: one patient underwent simultaneous major HNC surgery and died due to pneumonia on the 14th postoperative day. The other had collapsed and hit his head after being released from the hospital on the 5th postoperative day. He was found home unconscious, and resuscitation was not attempted because of a metastatic esophageal cancer.

## Discussion

To our knowledge, this is the first study to report patient demographics, indications, and operative details with follow-up for all adult consecutive tracheostomies performed by ORL-HN surgeons in one tertiary care center. Our department currently employs 55 otolaryngologists and 14 specializing otolaryngologists. During the study period, ORL-HN surgeons carried out 33% of all surgical tracheostomies performed for adults in the Helsinki University Hospital district, which is slightly more than that reported by Alfonso et al. (22%) [15].

Although we did not compare our procedures to those performed by other surgeons, we share the conclusion of Alfonso et al. that more challenging patients and complex tracheostomies are referred to otolaryngologists. ORL-HN surgeons often coordinate the treatment and perform the tracheostomies for HNC patients, who may have distorted anatomy due to the disease itself or because of prior surgical treatment. Furthermore, otolaryngologists frequently manage upper airway emergencies. Managing a difficult airway demands teamwork between ORL-HN surgeon and anesthesiologist and requires careful planning to ensure the best option for a secured airway. Emphasizing training during residency is crucial to confirm the competence of otolaryngologists while encountering challenging tracheostomies. We seem to be training our residents properly, as they comprise only 20% of employees at our department, but performed 37% of the procedures.

The most common indication for tracheostomy was simultaneous major HNC surgery. This finding differs from some of the previous studies, where the most encountered indication was prolonged mechanical ventilation [10–12]. However, these studies are conducted at intensive care units, hence the study population differs from ours. Nevertheless, Costa et al. studied ORL-HN surgeons´ urgent tracheostomies and reported HNC as the most common indication (45%), and deep neck infection the second (20%) [16], which concurs with the present study. Trauma, on the other hand, was a rare indication in our cohort. At our institution, all patients with a severe trauma are referred to a distinct trauma center, which is not led by ORL-HN surgeons, which can partly explain the low frequency of patients with trauma. Interestingly, Costa et al. [16] found only one (2%) tracheostomy indicated by supraglottitis, while we reported over three times more. There is still no clear consensus on the management of the airway in epiglottitis or supraglottitis, and fiberoscopic intubation might serve as an option for tracheostomy in selected cases.

A significant proportion of patients had high ASA scores, but low ACE-27 scores. ASA scores categorize patients’ overall physical status preoperatively, while ACE-27 is used to identify and classify the severity of their medical comorbidities. It seems that patients in our cohort suffered commonly from compromised airway that increased the ASA score, although they did not have significant comorbidities. However, comorbidity is reported to be related to survival, quality of life, and functional outcome in HNC patients [17]. The aspect of survival is comparable to our study, where all four deaths were encountered in patients with high comorbidity scores.

The time spent tracheostomized remained short for patients who were decannulated during the follow-up period. At the end of the follow-up, over one-third of the patients remained tracheostomy dependent, which is related to the high number of HNC patients in our cohort. Costa et al. [16] reported even higher dependence figures of 49%, and longer average cannulation periods of 4 months, and none of their HNC patients were eventually decannulated. The treatment for HNC is individualized, thus the optimal timing of decannulation or decision of a permanent tracheostomy is evaluated on a case by case basis.

Our 30-day mortality rate was 2%, which is significantly less than that reported by Kashlan et al. (16%) [18]. Their study concluded that comorbidities increased significantly the risk of death. Furthermore, in that study the main indication for tracheostomy was ventilator-dependent respiratory failure in contradiction to the indications in the present study. In our study, two deaths (1%) were related to tracheostomy, and it underlines the importance of patient selection and the operative team’s extra vigilance with patients with previous HNC treatment.

Our study includes limitations, which are mainly due to its retrospective nature. However, we were able to collect data on all tracheostomies performed in a tertiary care center covering over 3 years, enabling a large sample size with a minimum follow-up time of 18 months. Current literature provides limited knowledge on the indications, details of the surgery, and profile of the patients tracheostomized by ORL-HN surgeons. Thus, we believe our study will provide a new perspective on this heterogenic group. We found surgical tracheostomy to be safe, but it is of utmost importance that the whole team is well prepared and work in strict cooperation to manage these patients.

## Conclusion

Head and neck oncological surgery is the most prevalent indication for tracheostomy performed by ORL-HN surgeons. Nevertheless, almost two-thirds of the patients were decannulated during follow-up. The procedure was found safe; however, the cooperation of the surgical team patient selection are essential for avoiding risks.
